# 1651. Utility of *Staphylococcus aureus* Nares Screening in Hospitalized Children with Osteomyelitis

**DOI:** 10.1093/ofid/ofad500.1485

**Published:** 2023-11-27

**Authors:** Ashley Sands, Denise E Iacono, Stefan Hagmann

**Affiliations:** Cohen Children's Medical Center, New Hyde Park, New York; Cohen Children's Medical Center, New Hyde Park, New York; Hackensack Meridian Health, Hackensack, New Jersey

## Abstract

**Background:**

Antibiotics with activity against methicillin-resistant *Staphylococcus aureus* (MRSA) are frequently started empirically in children diagnosed with osteomyelitis. At our institution clinicians increasingly obtain at time of admission a PCR-based staphylococcal nares screen (SNS) which can differentiate methicillin-sensitive *Staphylococcus aureus* (MSSA) and MRSA. We aimed to evaluate the utility of SNS for antibiotic optimization in pediatric inpatients with osteomyelitis.

**Methods:**

A retrospective chart review was conducted of children (< 20 years) admitted for osteomyelitis (January 01, 2021 to December 31, 2022) who had a SNS and a clinical culture (i.e. site of infection or blood) performed as part of their diagnostic work up.

**Results:**

A total of 36 children were included, median age (range) of 8.5 years (0-19). Etiologic diagnosis was established in 52.8% (n=19), most infections were with MSSA (n=12), followed by MRSA (n=4), and MRSA/MSSA (n=1). The prevalence of nasal MRSA and MSSA colonization was 8.3% (n=3) and 47.2% (n=17), respectively. The sensitivity (SE), specificity (SP), positive predictive value (PPV) and negative predictive values (NPV) of the SNS test to predict a MRSA and a MSSA infection were 40.0%, 96.8%, 66.7%, 90.9%, and 75.0%, 66.7%, 52.9%, 84.2%, respectively. When including only patients with site specific cultures (n=25), SE, SP, PPV and NPV of the SNS test to predict MRSA and MSSA infection were 40%, 100%, 100%, 86.9%, and 70%, 60%, 53.8%, 75.0%, respectively. Of those whose SNS test was negative (n=16), infection with MSSA was found in some (n=3) but none had documented MRSA infection.

In about half (15/32, 46.9%) anti-MRSA therapy was discontinued during the hospitalization, de-escalation solely in response to a negative MRSA SNS occurred in some (n=3, 20.0%).

**Conclusion:**

We found that children with osteomyelitis have a disproportionate high rate of nasal colonization with MSSA which was also the primary etiologic cause for the infection in this cohort. The SNS was noted to show a high NPV for MRSA infection which may help encourage clinicians to de-escalate potentially toxic anti-MRSA therapy in a timely fashion, especially in non-critical patients. Further prospective study is warranted.

**Disclosures:**

**All Authors**: No reported disclosures

